# The complete chloroplast genome of *Syzygium acuminatissimum*

**DOI:** 10.1080/23802359.2020.1847615

**Published:** 2021-01-13

**Authors:** Feng Zeng, Yanwen Deng, Xiaozhou Liu, Xuanxi Zhu, Guangwen Tan

**Affiliations:** R&D Center, Pubang Landscape Architecture Co., Ltd, Guangzhou, China

**Keywords:** *Syzygium acuminatissimum*, chloroplast genome, phylogeny, Myrtaceae

## Abstract

*Syzygium acuminatissimum* is a valuable hard wood species in southern China. In this study, we sequenced, assembled and annotated the complete chloroplast genome of *S. acuminatissimum*. The complete cp genome of *S. acuminatissimum* was 159,352 bp in length, with a total of 109 unique annotated genes, including 78 protein-coding genes, 27 tRNA genes and 4 rRNA genes. Phylogenetic analysis showed that *S. acuminatissimum* was closely related to its congener *S. aromaticum*.

## Introduction

*Syzygium acuminatissimum* (Blume) Candolle, belonging to Myrtaceae, is an evergreen tree distributed in the low-latitude subtropical forests of China (Peng and Wang 1995). With perfectly straight trunk and hard wood, it is a valuable material for furniture (Zhou 2001). Previous authors studied leaf photoprotection and cultivation of this species (Liu et al. 2013; Zhu et al. 2018), and phylogenetic position of *Syzygium* in the family Myrtaceae was resolved based on sequences of three chloroplast (cp) fragments (Biffin et al. 2006). In this study, we assembled and annotated the complete cp genome of *S. acuminatissimum*, and investigated its phylogenetic relationship with related species in this family.

Fresh leaves of an *S. acuminatissimum* individual were collected from the Conghua Baimu Nursery (113°24′06″E, 23°43′04″N), Guangzhou and the voucher specimen (Zhang-20200729) was deposited at the Herbarium of Sun Yat-sen University (SYS), Guangzhou, China. Genomic DNA was extracted using the CTAB method (Doyle and Doyle 1987), and a DNA shotgun library was constructed according to the manufacturer’s protocol (reads size: 250 bp) and sequenced on an Illumina HiSeq X TEN platform (Illumina, San Diego, CA, USA). After quality control with SOAPnuke (Chen et al. 2018), about 2 Gb of clean reads was used to assemble the complete cp genome using SPAdes v3.13.0 (Bankevich et al. 2012), with the cp genome of *Syzygium cumini* (MN095412, template size: 1,58,499 bp) as the reference. Gene annotation was conducted by GeSeq (Tillich et al. 2017). The annotated cp genome sequence was submitted to GenBank (accession number: MT975437).

**Figure 1.**
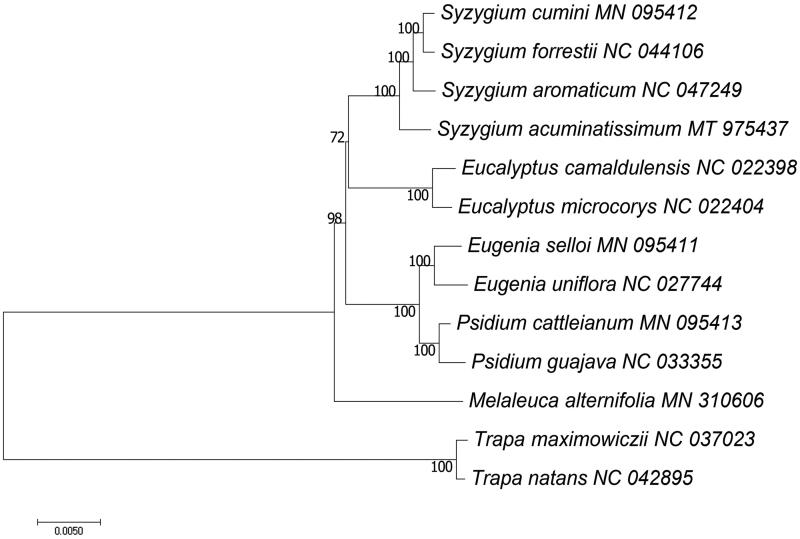
Maximum likelihood tree of 11 Myrtaceae species based on sequences of 78 common chloroplast protein-coding genes. Two Lythraceae species were used as outgroups. Bootstrap support values (%) are shown on branches.

The complete cp genome of *S. acuminatissimum* was 1,59,352 bp in length with a typical quadrant structure, including a large single-copy (LSC) of 87,993 bp, a small single-copy (SSC) of 18,417 bp, and a pair of inverted repeats (IRs) of 26,471 bp. A total of 109 unique genes, including 78 protein coding genes (PCGs), 27 tRNAs and 4 rRNAs, were annotated. The overall GC content of the cp genome was 34.73%. To resolve the phylogenetic relationships of *S. acuminatissimum* with other species in Myrtaceae, we selected 11 species from Myrtaceae as ingroups and 2 species from Lythraceae as outgroups. Based on 78 common PCGs, we used RAxML (Stamatakis 2014) to construct maximum likelihood (ML) tree with 1000 bootstrap replicates. The phylogenetic tree showed that *S. acuminatissimum* was closely related to *S. aromaticum* (Figure 1). The cp genome of *S. acuminatissimum* in this study provides useful information for future phylogenetic research on this genus.

## Data Availability

The data of this study are available on GenBank with the accession number MT975437 (https://www.ncbi.nlm.nih.gov/nuccore/MT975437).

## References

[CIT0001] Bankevich A, Nurk S, Antipov D, Gurevich AA, Dvorkin M, Kulikov AS, Lesin VM, Nikolenko SI, Pham S, Prjibelski AD, et al. 2012. SPAdes: a new genome assembly algorithm and its applications to single-cell sequencing. J Comput Biol. 19(5):455–477. 22506599 10.1089/cmb.2012.0021 PMC3342519

[CIT0002] Biffin E, Craven LA, Crisp MD, Gadek PA. 2006. Molecular systematics of Syzygium and allied genera (Myrtaceae): evidence from the chloroplast genome. Taxon. 55(1):79–94.

[CIT0003] Chen Y, Chen Y, Shi C, Huang Z, Zhang Y, Li S, Li Y, Ye J, Yu C, Li Z, et al. 2018. SOAPnuke: a MapReduce acceleration-supported software for integrated quality control and preprocessing of high-throughput sequencing data. Gigascience. 7(1):1–6. 10.1093/gigascience/gix120 PMC5788068 29220494

[CIT0004] Doyle JJ, Doyle JL. 1987. A rapid DNA isolation procedure for small quantities of fresh leaf tissue. Phytochem Bull. 19:11–15.

[CIT0005] Liu J, Huang W, Zhou G, Zhang D, Liu S, Li Y. 2013. Nitrogen to phosphorus ratios of tree species in response to elevated carbon dioxide and nitrogen addition in subtropical forests. Glob Chang Biol. 19(1):208–216. 23504732 10.1111/gcb.12022

[CIT0006] Peng S, Wang B. 1995. Forest succession at Dinghushan, Guangdong, China. Chin Sci Abstracts Ser B. 14:35–36.

[CIT0007] Stamatakis A. 2014. RAxML version 8: a tool for phylogenetic analysis and post-analysis of large phylogenies. Bioinformatics. 30(9):1312–1313. 24451623 10.1093/bioinformatics/btu033 PMC3998144

[CIT0008] Tillich M, Lehwark P, Pellizzer T, Ulbricht-Jones ES, Fischer A, Bock R, Greiner S. 2017. GeSeq–versatile and accurate annotation of organelle genomes. Nucleic Acids Res. 45(W1):W6–W11. 28486635 10.1093/nar/gkx391 PMC5570176

[CIT0009] Zhou T. 2001. Cultivation techniques of main economic trees in Tropical China. Beijing: China Forestry Press. p. 292–294.

[CIT0010] Zhu H, Zhang T-J, Zheng J, Huang X-D, Yu Z-C, Peng C-L, Chow WS. 2018. Anthocyanins function as a light attenuator to compensate for insufficient photoprotection mediated by nonphotochemical quenching in young leaves of *Acmena acuminatissima* in winter. Photosynt. 56(1):445–454.

